# “You're listening but you're not hearing”: qualitative exploration of parents' lived experience of paediatric sepsis

**DOI:** 10.3389/fped.2025.1655224

**Published:** 2025-09-15

**Authors:** Meagan O’Keefe, Liz Crowe, Paula Lister, Luregn J. Schlapbach, Megan Simons

**Affiliations:** ^1^ Queensland Paediatric Sepsis Program, Children’s Health Queensland, Brisbane, QLD, Australia; ^2^ Social Work Department, Queensland Children’s Hospital, Children’s Health Queensland, Brisbane, QLD, Australia; ^3^ School of Nursing, Midwifery and Social Work, The University of Queensland, Brisbane, QLD, Australia; ^4^ Wellbeing Department, The Royal Brisbane and Women’s Hospital, Brisbane, QLD, Australia; ^5^ Children’s Health Research Centre, Faculty of Health, Medicine and Behavioural Sciences, The University of Queensland, South Brisbane, QLD, Australia; ^6^ School of Medicine, Griffith University, Brisbane, QLD, Australia; ^7^ Department of Intensive Care and Neonatology, and Children’s Research Center, University Children’s Hospital Zurich, University of Zurich, Zurich, Switzerland; ^8^ Paediatric Intensive Care Unit, Queensland Children’s Hospital, Children’s Health Queensland, Brisbane, QLD, Australia; ^9^ Occupational Therapy Department, Queensland Children’s Hospital, Children’s Health Queensland, Brisbane, QLD, Australia

**Keywords:** sepsis, child, paediatric, lived experience, family, parent, family-centred, hermeneutics phenomenology

## Abstract

**Background:**

Sepsis is a life-threatening condition of significant mortality and morbidity burden in children. There is limited evidence exploring the lived experiences of parents whose children have survived or died from sepsis, nor their support needs, despite growing awareness of poor sepsis outcomes.

**Objective:**

This study aims to understand the lived experiences of parents of children affected by sepsis, both surviving and bereaved, and identify gaps in support services to inform future interventions.

**Methods:**

A phenomenological-hermeneutic design was used, with data collected through a focus group and individual interviews with 11 parents of children treated for paediatric sepsis in Queensland, Australia. Reflexive thematic analysis was used to analyse the data.

**Results:**

Four themes were generated that present an in-depth understanding of parents' lived experiences of having a child with sepsis and what their support needs are: 1. *Rupture of Life as We Knew It*—parents experienced a distinct rupture between life before and after their child's sepsis diagnosis, often accompanied by trauma and grief; 2. *Navigating in the Dark*—parents described feeling confused and isolated, driven by a lack of awareness about sepsis, inadequate communication from healthcare providers, and limited access to specialised support; 3. *The Weight of ‘What If’*—guilt, regret and disillusionment with the healthcare system were common among parents, particularly regarding missed early warning signs and opportunities for intervention; and 4. *Call for Change*—parents advocated for improved public and professional education about paediatric sepsis, trauma-informed communication from healthcare professionals, and the development of structured, specialised support networks.

**Conclusion:**

Parents of children affected by sepsis experience profound distress and isolation, compounded by a lack of awareness, inadequate communication, and limited specialised support services. They identified that urgent action is needed to develop paediatric sepsis-specific family support networks, enhance public and clinical education, and implement trauma-informed care to reduce the burden of sepsis on all families.

## Introduction

Paediatric sepsis has been recognised as a global health priority (1–5), with increasing investment in research and clinical outcomes through national and international collaborations (6–10). Yet, the voices of families, both those whose children survive and those who are bereaved due to sepsis, are largely absent from the evidence base, leaving a critical gap in understanding what families themselves identify as priorities for care and support.

Sepsis is a life-threatening condition caused by infection leading to organ dysfunction (6, 7). The Global Burden of Disease Study estimated 25 million cases of paediatric sepsis in 2017, of whom 3.4 million died (3, 11). Sepsis in childhood poses a significant mortality and morbidity burden. In Australia, approximately 60 children are estimated to die from sepsis each year (9), and one-third of paediatric sepsis survivors experience long-term morbidities across functional, cognitive, psychological and psychosocial domains which significantly impacts the child and their family (12, 13).

In 2017 the World Health Assembly published a resolution to improve the prevention, diagnosis, and management of sepsis (1). Australia was an early adopter and published “Stopping Sepsis: A National Action Plan” (2). Two of the four recommendations of the plan relate to investment in prevention and awareness campaigns to prompt community action and support services for sepsis survivors and their families. Working in partnership to design these campaigns and services is emphasised, stating “people recovering from sepsis and the families of patients who did not survive, remain the biggest and most important advocates for public awareness of sepsis, and it is essential they are directly involved and consulted in the design of community and peer support groups and services” (2).

There is a growing focus on understanding the short and long-term impacts of sepsis on surviving children (4, 12–14). However, to date there is an absence of the voices of families who have lived experiences of children who have had sepsis, their support needs, and perceived gaps in service. There is also a paucity of paediatric sepsis-specific consumer support processes in Australia (4). This critical gap has delayed the provision of holistic family-centred care to children and families affected by paediatric sepsis.

The aim of this study was to develop an in-depth understanding of the lived experiences of parents whose child has had sepsis (both surviving and bereaved). The objectives were: 1. to identify parental needs throughout the entirety of their child's sepsis experience (including the acute illness, hospitalisation, treatment, rehabilitation, discharge or into bereavement); and 2. inform future priorities for prevention, intervention and service design, to reduce the burden of sepsis on children and their families.

## Materials and methods

### Study design and ethical approval

This work was nested in the planning and early phase of a statewide quality improvement initiative in the state of Queensland, Australia, targeting sepsis across all age groups. Details on the Queensland Sepsis Collaborative have been reported elsewhere (8, 15). The present study explored the lived experience of parents and primary caregivers whose child had sepsis using phenomenological-hermeneutic methodology (16, 17), with reflexive thematic analysis (18) for data analysis. Hermeneutic phenomenology is a qualitative research methodology focused on understanding and interpreting the lived experiences of individuals, with an emphasis on how people make meaning of their experiences within their social and cultural contexts. It acknowledges the researcher's active role in co-constructing meaning through reflective engagement with participants' accounts (16, 17). Reflexive thematic analysis is a method for identifying and interpreting patterns of meaning across qualitative data. It emphasises the active role of the researcher in theme development and considers subjectivity an analytic resource when approached through ongoing reflexive practice (18). Ethical approval was granted from the Children's Health Queensland Hospital and Human Research Ethics Committee HREC/18/QRCH/173. The study was supported by a Children's Hospital Foundation grant (Grant Reference Number 50230); the funder played no role in the research. The results are reported using the Standards for Reporting Qualitative Research (SRQR) (19) (see Supplementary File S1).

### Setting, recruitment and participants

This study was conducted using an inclusion criterion of parents or caregivers (hereinafter referred to as “parents”) of children who:

1. spoke English and;
2. had a confirmed diagnosis of sepsis and;
3. received treatment in the state of Queensland.
4. Treatment sites included the Paediatric Intensive Care Unit (PICU) at Queensland Children's Hospital (QCH), Brisbane, Australia (from November 2014) or the former Royal Children's Hospital or Mater Children's Hospital, Brisbane, Australia (these sites merged in 2014 to become QCH) or;
5. another Queensland Hospital and Health facility and expressed interest in involvement in paediatric sepsis research.

Purposive sampling was undertaken to recruit parents with the requisite lived experience and knowledge of having a child with sepsis. Parents who had surviving children or were bereaved due to sepsis were included. To ensure diversity and relevance, the research team (MOK, LC, LS) used a combination of strategies to identify potential participants, including review of an internal sepsis database, and their own professional knowledge and recollection of families with whom they had previously worked. The researchers were cognisant of equity and inclusion and contacted a diverse sample of Queensland families from metropolitan, regional, rural and remote locations, and with varied ethnic and cultural backgrounds. Invitations were to both mothers and fathers, including where there were separated, divorced or blended families.

Parents were telephoned by the Principal Investigator (MOK), or a hospital Social Worker known to the family, to provide the parents with an overview of the research. Parents who expressed an interest in participating were mailed an information pack containing details about the research and an invitation to participate. Those who chose to participate were informed they could choose between a semi-structured, audio-recorded, face-to-face focus group, or an individual telephone interview with the Principal Investigator (MOK). Parents had autonomy in deciding whether they participated in the single focus group or the individual interviews. Written consent was obtained prior to participation. Parents were aware that they could withdraw from the study at any time.

The research team have extensive clinical backgrounds in paediatric critical care and the care of families impacted by paediatric sepsis, as well as research experience. The research team included two experienced PICU Social Workers with decades of experience working with families affected by critical illness and bereavement (MOK, LC), two paediatric intensive care specialists with expertise in paediatric sepsis (PL, LS) and two clinician-researchers with extensive experience in qualitative health research (MS, LC). Two authors (MOK and LC) had established clinical relationships with many of the families contacted, which raised important considerations regarding the influence of these prior relationships on the research process. The practice of constant reflexivity was employed throughout data collection and analysis to ensure their working knowledge of the field of study, and prior professional knowledge of some participants deepened the exploration of participant's lived experience (20, 21). The research team reflected on power dynamics, positionality, and the potential impact of these factors throughout the study and made this transparent with participants. The Principal Investigator (MOK) documented thoughts, feelings and areas of interest after the focus group and each interview. Regular peer debriefing with the Co-Investigator (LC) and research team further facilitated awareness of predisposed beliefs, values, assumptions and experience brought into the research process. Many of the parents expressed deep gratitude for the opportunity to tell their stories and advocate for their rights and improvements within the system. One bereaved parent shared that inclusion in the study was “*like someone turning on the light after five years of sitting in the dark” (bereaved parent 2*).

### Data collection

A single focus group was conducted with the Principal (MOK) and Co-Investigator (LC) at the Queensland Children's Hospital, Brisbane, Australia, in September 2018. Individual interviews were conducted between September and December 2018 with the Principal Investigator (MOK) via the telephone. A semi-structured interview protocol was developed for the focus group and individual interviews (see Supplementary File S2). The duration of the focus group was 148 min, and most individual interviews lasted 50 min (range between 25 and 72 min). The focus group and individual interviews were audio recorded, transcribed verbatim and de-identified prior to further analysis.

Given the potentially distressing nature of the topic, the team's clinical expertise was critical to ensuring ethical engagement. The focus group and interviews were conducted by PICU Social Workers (MOK, LC) who were highly experienced in supporting families through trauma and grief. This clinical expertise enabled the researchers to recognise and respond to the signs of participant distress, ensure a safe and compassionate interview environment, and offer debriefing and follow-up support where needed. The Co-Investigator (LC) recorded field notes during the focus group documenting contextual information including non-verbal interactions between participants. The Principal Investigator (MOK) documented field notes during the individual interviews.

Interviews were ceased when all consenting participants had participated in either an individual interview or the focus group. Data saturation was not used to guide sample size. Instead, consistent with the exploratory nature of the study and its hermeneutic phenomenological methodology, all participants who responded to the invitation were included to prioritise depth and richness of individual experience (16, 17).

### Data analysis

Braun and Clarke's reflexive thematic analysis method was used for inductive analysis of the qualitative dataset (18). Analysis was conducted manually without the use of qualitative data analysis software, allowing the researchers to remain closely engaged with the data throughout the coding and theme development process. Familiarization with the data was achieved by the Principal Investigator (MOK) and Co-Investigator (LC) independently reviewing the audio and transcripts at least twice, to develop a sense of the whole and begin exploring patterns, commonalities, and atypical cases in the data. Initial codes were independently generated from all interviews. The investigators (MOK, LC) categorised the codes based on differences and similarities. Finally, the underlying meaning of the categories were refined into themes in discussion with the research team. Rigor and trustworthiness were established through multiple strategies. Credibility was supported by the involvement of multiple investigators in data collection, coding, and interpretation (investigator triangulation), by inviting participants to reflect on and respond to key themes (member checking), and through immersive engagement with the data (22, 23). These strategies were chosen to increase the richness and diversity of perspectives and maintain a dialogical relationship with participants to invite further reflection that could inform the interpretive process (24). Dependability was strengthened through peer debriefing and reflexive journaling. Transferability was enhanced through purposive sampling across diverse geographic and demographic contexts, and by providing rich, thick descriptions of participants' experiences.

## Results

Twenty-one parents of 19 children were contacted. Eleven parents of 10 children consented to participate. All participants were the biological parents of their children. Two fathers participated. Bereaved parents had a higher rate of participation in the study (7/11), and most families were from metropolitan or regional locations (8/11). The children of the participants were treated for sepsis between 2003 and 2017, however most of the children experienced sepsis within two years of the study (7/11). Nearly all children (10/11) were treated in the PICU, with the exception of one child who died in the emergency department before transfer to the PICU could occur. The median age of children at the time of sepsis diagnosis was 4 years (with a range of 9 months to 16 years). The 11 parents were representative of children impacted by sepsis in Queensland in that they identified as either Caucasian, Aboriginal or Torres Strait Islander peoples or Polynesian and lived in rural, regional, and metropolitan areas. Other demographic details are not reported to maintain confidentiality. Ten families declined to participate. Those who declined differed from the participants in that the children were older, and most of the children had survived sepsis. Between September and December 2018, one face-to-face focus group (with 8 participants, of which 5 were bereaved parents) and three individual interviews (3 participants, of which 2 were bereaved parents) were conducted.

Thematic analysis of the focus group and interview data generated four themes that offer insight into the lived experiences of parents of a child with sepsis, their support needs, and their priorities for future interventions: 1. Rupture of Life as We Knew It; 2. Navigating in the Dark; 3. The Weight of ‘What If’; and 4. Call for Change. No meaningful differences in themes were identified between the focus group and individual interviews. Through the member-checking process, all participants indicated that the four themes resonated with them and authentically represented their lived experiences of paediatric sepsis. See Supplementary File S3 for a more detailed presentation of illustrative comments.

### Rupture of life as we knew it

Every parent described an exact moment when life changed due to sepsis; whether it was a specific date, the diagnosis, a sudden deterioration, or the moment their child died. They spoke of a distinct rupture between life before and after sepsis. This shift was described through painful dichotomies: the healthy child vs. the critically unwell one, familiar childhood illness vs. the unknown threat of sepsis, and the once-predictable future now fractured by trauma and loss.

Many parents reflected on their assessment of their child's condition. They referred to how their condition changed subtly from what they believed to be a familiar childhood illness, of which they assumed the child would recover, to an instinctual feeling that *something was different*. Participants reported recognising that something about their child had changed, even if they were unable to articulate it. As one parent described:

“My husband thought he was resting and getting better. But…I said, ‘something’s wrong, that’s not right’. So, we called the after-hours [medical service] and, then it was all on, the ambulance came, and they took him in.”—Bereaved Parent 5

“I said to my husband ‘it’s just not right, something’s just not right’.”—Survived Parent 1

Throughout the time that their child was critically unwell, parents were continuously preparing for multiple potential life trajectories reflecting the varying morbidities that their child was facing. For example:

“And they started to warn me that he might lose limbs, he might need this, he might need that and was trying to prepare us for if he survives.”—Bereaved Parent 1

Both bereaved parents and parents of children who survived found it important to tell the story of who their child was before developing sepsis, particularly in terms of physical appearance and overall health. All the participants expressed continued bewilderment that their healthy, robust children had become critically unwell and visually confronting due to medical interventions, physical changes to their bodies and/or having died. Bereaved parents reflected that how their beautiful, healthy, happy children had lived their lives was in stark contrast to the horrible, sudden, and distressing deaths they experienced.

“My son was a very robust, big, seemingly healthy child…and eight hours later we lost him. Cardiac arrested three times…it was too late.”—Bereaved Parent 1

Sepsis shattered families' worldview. The parents reported that everything they trusted to be true and predictable about their child's health and safety (and that of their other or future children) ceased to exist when their children became critically unwell or died from sepsis. They expressed shock and fear that children could become so rapidly and life-threateningly unwell from a condition they had limited understanding of. As one parent stated:

“You’re gripping on to, you know, babies don’t die, not in 2018, not in Australia.”—Bereaved Parent 2

### Navigating in the dark

Parents described feeling unprepared and overwhelmed as they navigated their child's sepsis journey, often with limited understanding and support. Inconsistent care, rapid deterioration, and minimal information left families confused and isolated.

Parents reported that they were largely unaware of sepsis prior to their child becoming unwell. All participants spoke about the fear of negotiating the unfamiliarity of sepsis, medical terminology, and interventions.

“In our situation, yeah, completely naïve to the word ’sepsis’. And, um, my husband had studied one year of medicine.”—Bereaved Parent 2

Many of the parents described that during the acute phase of the sepsis illness they had tried on multiple occasions to seek help for their child from an array of health professionals and health systems, and they experienced inconsistent responses. For many, following an initial presentation, they were encouraged to return home. Most participants described receiving minimal or no safety plan, little to no education on serious illness or encouragement on when or if to re-present if they remained concerned. One parent recalled:

“She [daughter] sort of tried to stand on her legs, and she couldn’t. The doctor called another doctor in and um, didn’t really give me much interest. I think the general comment was ‘oh, you know it’s Influenza, we’ve had a lot of that this season, just go home and rest’.”—Survived Parent 1

Parents reported receiving limited information on sepsis from clinicians when their child was critically unwell. The limited information that parents did receive was provided verbally, with no consideration to the fact that their capacity to process information was adversely affected by stress and trauma. This resulted in parents having a collective difficulty comprehending the severity of their child's condition, such as:

“It’s just a blur like nobody explained what it could be. We didn’t, I didn’t comprehend how sick she was at all.”—Bereaved Parent 3

“You’re listening but you’re not hearing.”—Bereaved Parent 5

The deterioration of children with sepsis was universally experienced as rapid, visual, and shocking. Within hours of admission to hospital, many of the children were mechanically ventilated, or on extra-corporeal membrane oxygenation (ECMO) support or deceased. Parents identified they were, and remain, shocked and traumatised by how quickly their child deteriorated. They reported their distress was amplified by their limited understanding of sepsis and where to seek information and support. Parents described that this distress had extended far beyond the acute illness of their child.

“At the time I still thought he would live, I didn’t think he was going to die. I even said, ‘Is my baby going to die?’ [Nurse replied,] ‘No sweetheart, your baby is not going to die, he has improved, he is getting better’. And then I shouted, ‘Somebody needs to look at my baby, somebody help me’ [crying, gesturing as if holding child]. He died.”—Bereaved Parent 2

“I was walking back, and I heard them saying her heart had stopped. I remember just standing there in ICU and I yelled at my husband ‘we are losing her’ and they were pumping her chest.”—Survived Parent 1

Parents described the PICU as an overwhelming and unfamiliar environment. Many spoke of the practical challenges they faced, such as not knowing where basic facilities were located, which added to their sense of disorientation and distress.

“You want your family close, and you need support but there’s nowhere to eat, or sleep, or where are the toilets if you’ve never been in a hospital situation, where can I shower?”—Survived Parent 1

All the participants continued to report finding sepsis a complex and difficult condition to understand and define. This resulted in feelings of confusion and fear that their surviving child could develop sepsis again. Parents expressed anxiety that, even having been through the experience once, they might still be unable to recognise the signs and symptoms if sepsis were to occur again. Parents also expressed concern about the safety of siblings and future family planning:

“But I was talking with other people about it, about whether or not I think I’d recognise it again. And I like to think that I could, but maybe I wouldn’t, you know?”—Bereaved Parent 4

Limited awareness of sepsis in the community and the lack of sepsis-specific resources caused participants to feel isolated and unable to seek help from their usual community support networks of family and friends. Parents stated they blindly searched online for support groups and information on the long-term impacts of sepsis in survivors. As one parent shared:

“Because sometimes at home I feel like nobody really understands what we went through, or what sepsis is. And I’ll just be driving with him down the road and I’ll remember what happened to him and absolutely bawl my eyes out.”—Survived Parent 3

Families living in rural and remote areas experienced isolation more acutely.

“There’s not even any child loss support groups in my [hometown] let alone sepsis child loss support groups.”—Bereaved Parent 7

The bereaved parents perceived that losing their child from sepsis was unique, complex and isolating. There was a connection and sense of relief between the bereaved parents to know that other parents had similarly felt the guilt, anger and disillusionment with the health system following their child's death from sepsis.

“I think what I found was after we went home, and after everyone else left, I’ve never felt so isolated, and I felt like I was the only parent in the world who has lost a child to sepsis. And I went online looking for groups, or other bereaved parents and I couldn’t find anything.”—Bereaved Parent 3

The majority of bereaved parents reported that their grief and trauma was amplified when they were informed of any coronial process and/or postmortem examination required after their child's death. Most parents identified the formality of the coronial process as distressing, lengthy and confusing to navigate*.*

“Just before we were about to say goodbye they said, ‘they’re going to do an autopsy, the coroner will take over’. And they had to do an autopsy, and we just said no, the last thing this little girl needs is that.”—Bereaved Parent 3

### The Weight of ‘What If’

All the parents expressed being overwhelmed by profound and enduring regret, guilt, and anguish when reflecting on their child's sepsis diagnosis and treatment, irrespective of outcome.

Parents talked of constant rumination and fear that they as parents or that one of the health professionals may have missed an opportunity to change the course of their child's sepsis experience and outcome. Parents expressed how they continue to ruminate over their decisions and the dismissal of their parental instincts, leading to feelings of being tortured by the heavy weight of *what ifs*:

“That’s always in my mind and will never go away. If we had of done blood tests on that day, rather than waiting to the next day to go to the hospital, would the outcome have been different? And the outcome is what it is. But that’s one thought I have struggled with.”—Bereaved Parent 3

“But I have guilt, it’s a stupid guilt, but I remember before we lost him, I whispered in his ear, ‘if it’s too hard [son’s name] you can go’. And I regret it, I said ‘what if I had told him to fight, would he have listened?’ Like, so that’s a regret I have, that I told him it was all right to go. Well sure enough on Friday he left. And I just thought, if anyone’s going to tell him he can go it’s his Mother. But I regret it, I still regret it, in a stupid way I regret it.”—Bereaved Parent 5

Participants shared instances of successfully advocating for their child throughout initial medical investigations and management. However, they also perceived multiple barriers in the healthcare system. These included a fear of “*annoying”* health professionals, or having parental concerns minimised because of the perceived expertise of health professionals. Many parents reflected on being in a vicious cycle of *what if*; *what if* they had remained confident in their knowledge of their child, *what if* they had advocated more fiercely by asking more questions, seeking second opinions, or having the knowledge to ask about sepsis or when to re-present. One parent reflected:

“She [the Doctor] said ‘go home and rest’. Okay you know, [I thought] ‘well they know what they are talking about, I don’t, off you go, we will go home’. We were so worried.”—Survived Parent 1

### Call for change

It was important for parents to find a sense of meaning and purpose after their child had become critically unwell, or died from sepsis, as they navigated this new and unfamiliar world. Parents voiced a powerful desire for learnings from their experiences to drive change and improve outcomes for other children and families navigating paediatric sepsis.

Participants gathered hope that other families might be spared similar experiences when clinicians learned from the clinical story of their child, or when they could participate in sepsis-related research, fundraising, and provide education about sepsis to health professionals and the public. For bereaved parents, these efforts to find meaning and create change represented an ongoing commitment to honouring their child:

“I think not long after he passed, they had quite a lot more success on putting some other kids on two machines [ECMO]. So, I feel a bit of joy in that. Maybe the opportunity to put him on that helped another couple of children.”—Bereaved Parent 4

Parents highlighted the importance of family-centred care in supporting their coping while in the emergency department or the PICU. This included compassionate end-of-life care; support of siblings; preserving their child's personhood; facilitation of extended family/friend's visitation; environment of care; regular and unambiguous communication; and access to practical support and facilities.

“We got to take him outside on the balcony, and… [pause, crying], I think it was four thirty in the afternoon, so the sun comes in, and he got to be outside to pass. So, we was appreciative of that. The extra effort that everyone here went to to help his Mum and myself and our other son.”—Bereaved Parent 4

The participants universally called for the urgent development of a paediatric, sepsis-specific, family support network. They reported that they would have benefited from sepsis-specific support through every stage of their sepsis crisis, including adjustment to life after sepsis or in bereavement. There was consensus from the parents that a range of services for support and information were necessary—including peer mentorship, educational resources, opportunities for community engagement, and avenues for advocacy. Parents stated that it was imperative that these services were co-designed with those with lived experience. All parents called for the development of a formalised group of consumers to oversee and inform paediatric sepsis-specific support structures, public health initiatives and research developments—ensuring that all efforts remain grounded in the needs, perspectives, and priorities of those they are intended to serve (such as a Consumer Advisory Group). As one parent stated:

“And you just want to talk, and your friends try and help, and your family tries to help, but you just want to talk to someone who just goes ‘I get it, I get it’. Even just sitting here, I feel like I’m not the only one, for the first time in two years.”—Bereaved Parent 3

The parents were passionate about the need to educate the public and health professionals on the signs and symptoms of sepsis, the need for immediate medical intervention and safety netting to attempt to ensure the safety of other children. Parents reiterated the importance of informing other parents to listen to their *gut instincts* as part of awareness campaigns and future education programs and resources.

“Trust your gut. You know your kid. If you know that there’s something wrong with your child do not leave that hospital.”—Survived Parent 2

Parents recommended that the advice given to families of children discharged home with an infection should include the importance of monitoring at home; including watching for signs of deterioration, lack of improvement or an instinctual feeling that “something is not right”. Parents emphasised the need for clinicians to empower parents to re-present if they have ongoing concerns.

“You might not have all of those symptoms because everyone has different symptoms, you might have some, but if you’re worried come back, you’re welcome to come back.”—Survived Parent 1

There was consensus from the participants that all parents would benefit from the option to receive multi-modal sepsis education (e.g., spoken, written, video) from the moment that sepsis is considered as a diagnosis. Parents identified the need for education on escalation pathways if they are feeling unheard or believed their child's condition was not being adequately managed.

“I think a pamphlet would have been good for me, like in that time when we were first in ICU, I might have gone back there [to the accommodation] in quiet times and looked at it then away from the bed. I think I would have liked to have known.”—Survived Parent 1

Bereaved parents and parents of children who survived sepsis identified their future trajectories as separate with unique ongoing needs. These two distinct groups respectfully acknowledged requiring separate support groups for the future to allow their unique needs to be met and validated.

“But the difference is that we’ve lost our children, our need is our grief and then to process that. We’ve all gone through a similar trauma. But where our life goes, your life, and your challenges now, they are just very different to what ours are going to be.”—Bereaved Parent 1

Families from rural and remote areas advocated for equitable access to resources including timely intervention and treatment of sepsis, education and support. As one parent stated:

“And I want to fly the flag for rural and regional Queensland too. It would be wonderful if, regardless of where you live in Queensland you felt safe.”—Bereaved Parent 2

The findings from this study have been summarised as clinical considerations (see Table 1) to support health service and healthcare providers to strengthen family support systems and reduce the burden of paediatric sepsis on children and their families.

**Table 1:** Clinical considerations for establishing a family-centred paediatric sepsis service.

Consideration	Details and examples of consideration
Education tools on sepsis signs and symptoms for all families	Increase families access to information on signs and symptoms of sepsis through advocating for public awareness campaigns, providing written resources in healthcare waiting rooms, visual posts on health social media pages etc.
	Include phrases “trust your gut” and “could it be sepsis?” in public awareness campaigns and education resources, highlighting parents' expertise of their child and their crucial role in early detection of sepsis.
Safety netting prior to discharge (when child assessed to not currently have sepsis)	Provide family with verbal, written and visual information detailing: - signs and symptoms of sepsis - sepsis being a medical emergency - role of parents in early recognition - encouragement for prompt re-presentation if their child's condition deteriorates, doesn't get better, or if they remain concerned
Informing families of available escalation pathways	At point of admission to hospital provide families with verbal and written information on escalation pathways available to them if they are concerned that their child is getting worse or not getting better, and they are not receiving appropriate responses from their medical team
Providing families with timely and trauma-informed information on sepsis	When sepsis diagnosis is considered or confirmed provide families with multi-modal information on sepsis and management plan. This can include verbal, written and visual information, provided regularly throughout acute illness and hospitalisation.
Clinician training	Clinicians engage in regular training and education on: - recognition and management of sepsis - parental concern as diagnostic tool - safety netting and escalation pathways - complex grief, trauma, and support needs of parents impacted by paediatric sepsis
Developing and facilitating a paediatric sepsis family support network	This structured network should focus on the education, connection and empowerment of families impacted by paediatric sepsis. This could involve: - facilitated and structured peer mentor program - access to sepsis education resources - development of sepsis education resources - opportunity for media engagement to raise sepsis awareness - participation in research focused on paediatric sepsis - connection with other families with shared experiences Inform parents of supports and resources available to them throughout the entirety of their child's sepsis experience, including into bereavement if their child died.
Providing specialised bereavement care	Develop and provide families with tailored bereavement care that recognises the unique experiences and support needs of parents whose child has died from sepsis. This could include: - Peer mentor program for bereaved families (separate to peer mentor program for surviving families) - Bereavement resources that acknowledge the complex grief and trauma experienced by families bereaved by sepsis. - Arranging in-person meet ups for families whose child has died from sepsis
Meeting needs of families living in rural and remote areas	Recognise and address the specific challenges faced by rural and remote families in accessing time-critical paediatric sepsis care and managing life after sepsis. Commit to developing flexible, accessible models of care and support that are responsive to the needs of this patient group across the continuum of care.
Development of culturally responsive support and resources	Prioritise genuine partnerships with community leaders within communities at higher risk of being impacted by paediatric sepsis such as Aboriginal and Torres Strait Islander communities, and Culturally and Linguistically Diverse communities. Ensure that support services and resources are culturally safe, responsive and shaped by community priorities.
Commitment to co-designing services	Partner with consumers to co-design all sepsis information and support materials. Ensure representation from vulnerable communities in these processes. Commitment to this process acknowledges and respects families lived experience, their expertise in their child, and their knowledge of what their education and support needs are.

## Discussion

This qualitative study explored the lived experiences of a sample of parents whose children were diagnosed with sepsis, encompassing perspectives across the illness trajectory for both bereaved parents and those whose children survived. Thematic analysis of the data from the focus group and interviews generated four key themes that capture how parents made sense of their experiences and what they needed to feel supported: 1. *Rupture of Life as We Knew It,* 2. *Navigating in the Dark,* 3. *The Weight of ‘What If’*, and 4. *Call for Change*.

Existing studies of the impact of paediatric sepsis largely focus on survivor's long-term physical, neurological, and cognitive morbidities, with limited research available exploring the psychosocial impacts (4, 12–14). There are no studies exploring the impact on bereaved families. Similar findings to this study were found in the Life After Pediatric Sepsis Evaluation (LAPSE) study which measured the distress and functioning levels of caregivers of surviving children after community-acquired paediatric septic shock using validated questionnaires (25). Although most families showed resilience, a subgroup exhibited elevated distress and dysfunction at 12 months, leading the authors to recommend proactive psychosocial screening and support. There is, however, no published exploration of the described lived experiences of parents covering the entirety of their child's sepsis experience in the literature. The primary novel contribution of this study is the widened lens exploring parents' lived experiences from the onset of their child's critical illness through hospitalisation, rehabilitation, survivorship, and into bereavement if their child died. This is also the first published study to identify and report on the support and intervention needs of families impacted by sepsis.

The lived experiences of the wider cohort of parents and carers of children receiving care in PICU for varied critical illnesses, not limited to sepsis, have been explored in several prior studies (26–29). The importance of family-centred care while in PICU mirrored the experiences of parents in the current study. Previous studies had a narrowed focus on the impacts of the PICU experience, whereas this study demonstrates parental lived experience of paediatric sepsis across the entire trajectory of the illness. With the exception of the importance of family-centred care, the findings of this study are specific to the sepsis diagnosis, and therefore not generalisable to the wider PICU cohort or other acute paediatric illness populations.

The theme of *rupture of life as we knew it* captured the profound rupture parents experienced as they transitioned from life with a healthy child to life marked by trauma, uncertainty, and irrevocable change; the stark dichotomy between “then” and “now”. This sudden and extreme contrast marked the collapse of their previous worldview—the taken-for-granted beliefs that the world is predictable, and previously healthy children do not suddenly become critically ill from an unknown condition. This aligns with Janoff-Bullman's (1992) concept of the “assumptive world,” where individuals' assumptions about safety and predictability are violently disrupted by trauma (30). Such violations lead to a profound disillusionment with the stability and predictability of life, which was echoed in the parents' accounts. This was particularly evident for those who experienced bereavement, where the death of a child from sepsis represented the ultimate violation of the deeply held assumption that children will outlive their parents (31). These findings contribute to the broader understanding of the psychological toll that sepsis takes on families, underscoring the long-term emotional burden, and highlight the need for targeted, sepsis-specific support services.

Participants described the experience of their child's critical illness with sepsis as being akin to *navigating in the dark.* Inconsistent communication from healthcare professionals compounded the uncertainty and confusion they experienced. Most participants were unaware of sepsis prior to their child's diagnosis and did not know of the extreme life- and limb-threatening nature of the condition, and the potential for rapid deterioration. These findings echo research demonstrating low sepsis awareness and understanding among the Australian public, particularly among parents of young children (32) and reinforce the urgent need for public health interventions. Importantly, this study adds new qualitative insight into how a lack of awareness is experienced by families in real-time, and how it contributes to confusion, fear, and potential missed opportunities for seeking early healthcare.

The profound and pervasive isolation parents described throughout the entirety of their child's sepsis journey is a new finding in the literature. Participants could not access support from their usual networks, because these communities had limited knowledge of sepsis and the trauma they had experienced. Despite considerable resilience and resourcefulness in seeking support, participants found a lack of sepsis-specific information and tailored services amplified their sense of isolation. For bereaved parents, this isolation was particularly striking—a novel insight in the context of sepsis. While the death of a child is universally isolating (33), participants described feeling uniquely alone because their child died of a condition few understood and for which no specialised bereavement supports existed. These findings expose an as yet undocumented gap in psychosocial care for sepsis-affected families and underscore the urgent need for targeted, condition-specific support networks to mitigate this isolation.

The theme *the weight of ‘what if’* expressed the enduring psychological toll experienced by parents as they ruminated on moments where earlier recognition or different actions, by themselves or clinicians, might have altered the outcome for their child. Parents often experienced repeated presentations to healthcare prior to their child's critical deterioration and had later knowledge that some of these encounters had responses from health professionals that were ill-informed. These experiences contributed to a dual sense of betrayal and self-blame. Parents internalised them, questioning their own judgement and feeling that their limited knowledge of both sepsis and the health system may have impeded their ability to advocate effectively. These findings offer new insights into the psychological burden parents carry when their early concerns are dismissed in the context of a rapidly progressing illness. Parents identified multiple barriers to seeking or re-presenting to healthcare and communicating their concerns for their child. These barriers were frequently underpinned by an internal conflict between deferring to medical expertise and trusting their parental knowledge that something was different about this illness in their child. This study builds on emerging literature recognising parents as experts in their child's baseline behaviour when presenting with severe infection (34–40) and highlights how this knowledge can be a critical early warning signal for sepsis.

Motivation to lead a *call for change* was strongly expressed by parents throughout the study; emphasising that learnings from their devastating experiences must lead to improvements for future families affected by paediatric sepsis (summarised in Table 1). Parents strongly advocated for the development of a structured, accessible, paediatric sepsis support network as a vital intervention for future families. Interventions must recognise the unique and complex experiences of families throughout the sepsis journey, from acute illness, through to survivorship and bereavement. Participants described a range of desired components which serve as emotional and practical resources and support, and importantly, as avenues for meaning-making and post-traumatic growth (41). Accessibility, irrespective of geographical location, was identified as essential. Australians living in rural and remote areas experience disparities in health outcomes compared to those living in metropolitan areas (42, 43). Participants called for greater visibility of rural and remote families' experiences in policy and service design. Importantly, participants highlighted that the needs of bereaved families are distinct to those whose children survived sepsis. Existing generic bereavement services are rarely tailored to the unique trauma and isolation that can accompany a sepsis-related death. Parents called for support services to be co-designed with those who have lived through the experience, to ensure that they are both relevant and responsive. These findings align with and add momentum to national recommendations such as those outlined in the *Stopping Sepsis: National Action Plan* (Recommendation 2a) (2), while providing new, lived experience insight into the specific features families deem essential in such a support network. By detailing the emotional, informational, and social needs of parents, this research offers a more nuanced roadmap for the development of family-centred, trauma-informed support in paediatric sepsis.

Building on Recommendation 2a of the *Stopping Sepsis National Action Plan* (2), the participants expressed the urgent need for targeted public awareness campaigns that educate the community about sepsis and promote early detection. Parents request the campaign validates parents' intuition and empowers them to advocate for their child's health with language such as “trust your gut” and “could it be sepsis?”. This emphasis on parents as active partners in early recognition builds on existing literature that identifies early recognition as critical, particularly given that most paediatric sepsis cases originate in the community and escalate rapidly within the first 48 h of hospitalisation (44–47).

Parents called for improved communication and information provision across all stages of the sepsis journey—including when a child is assessed to be unwell but not diagnosed with sepsis, and when sepsis is first suspected. They stressed the need for effective safety netting advice when a child is discharged with an infection, including clear, verbal, and written guidance on sepsis signs and symptoms, its rapid onset, and encouragement for prompt re-presentation if parental concern persists. This supports previous findings on the value of structured safety netting (36, 48) and aligns with broader recommendations such as those from the National Institute for Health and Care Excellence (NICE) (49–51). When sepsis is first suspected, participants described a need for timely and clear information about what sepsis is, its life-threatening potential, and the anticipated treatment plan. They emphasised that this communication must be trauma-informed, acknowledging that the acute crisis and psychological distress surrounding a sepsis diagnosis may significantly impair their ability to absorb and retain information (52). Participants recommended a multi-modal approach so that information could be revisited when they were ready. These findings underscore the need to equip healthcare professionals with both sepsis knowledge and communication skills to deliver consistent, accessible, and trauma-informed education at every stage of the sepsis journey.

The findings within the theme *call for change* align closely with the family-centred care principals of respect and dignity, information sharing, participation, and collaboration (53), which was identified as a priority in the International Guidelines for Management of Sepsis and Septic Shock (54).

Although data collection was completed in 2018, the findings of this study remain highly relevant to the current sepsis care landscape in Australia. There is growing national recognition of the importance of improving sepsis care, most notably through the release of the Australian Commission on Safety and Quality in Health Care's Sepsis Clinical Care Standard 2022 (5). In Queensland, the Queensland Paediatric Sepsis Program (QPSP) revised the clinical pathways in 2023 to align with the Sepsis Clinical Care Standard. Importantly, this study served as an impetus for the co-design of the QPSP Family Support Program (55) and contributed to a broader recognition of the importance of embedding lived experience in the design and implementation of sepsis care initiatives (56). While these developments signal positive change, they represent the early stages of a longer journey. Family support programs, public awareness campaigns, and information for families affected by sepsis are not yet routinely available across Australia, and there remains limited systemic focus on the longer-term impacts of paediatric sepsis beyond the acute phase. The central themes of this study highlight ongoing priorities for service design and delivery that require sustained attention and action.

### Limitations

The main limitations of this study relate to the sample. Families were contacted through a centralised large PICU in Queensland, Australia, and almost half declined consent. The research team used a combination of strategies to identify participants, including their professional knowledge and recollection of families with whom they had previously worked, which may have introduced selection bias. While efforts were made to ensure diversity, these sampling strategies should be considered when interpreting findings. Since this research, an organisation-wide clinical dashboard has been developed which reports prospectively collected inpatient admissions data which may reduce the risk of selection bias in future research.

Aboriginal and Torres Strait Islander children are disproportionately affected by sepsis due to systemic health disparities (10, 57–59), and only one Aboriginal parent participated in this study. Therefore, this study does not represent the voices and experiences of Aboriginal and Torres Strait Islander families, nor the in-depth exploration of culturally safe and responsive support planning that is required. Future research may enhance inclusion and representation of Aboriginal and Torres Strait Islander peoples by prioritising genuine partnerships with community leaders and Indigenous Health Workers.

The inclusion of only English-speaking participants limited the representation of culturally and linguistically diverse (CALD) communities. This is an important consideration, as families from CALD populations may face increased risk of delayed sepsis diagnosis and treatment due to socio-cultural and systemic barriers (60). These barriers, however, coexist with rich cultural knowledge, resilience, and strong community connections that greatly assist navigating the healthcare system. As a result, the unique experiences and perspectives of families from CALD populations may have not been adequately captured in this study.

Parents of children who survived sepsis or were further away from the time of their child's sepsis were less likely to agree to participate. Therefore, further research would be beneficial to explore the generalizability of these findings to families whose children survived sepsis, and those who are further away from the time of their child having sepsis.

Although reflexive thematic analysis allows for rich and in-depth engagement with the data, it is inherently shaped by the researchers' interpretations. Despite multiple strategies to enhance trustworthiness, there remains a risk that less prominent or more complex narratives and themes may have been underrepresented. To help mitigate this risk, participants were invited to reflect on and respond to the identified themes (member checking), and all participants who provided feedback reported that the findings resonated with their experiences.

## Conclusion

This study demonstrated the profound impact of sepsis on parental experiences, emphasising the significant distress and isolation they face due to limited awareness, rapid deterioration, inadequate communication, and insufficient specialised support services. By understanding the unique challenges of families affected by paediatric sepsis, healthcare providers and healthcare systems can implement targeted, family-centred interventions to improve both patient outcomes and parental wellbeing. Urgent action is required to establish paediatric sepsis-specific family support networks, enhance public and clinical education, and integrate trauma-informed care into practice to reduce the burden of paediatric sepsis on all families.

## Data Availability

The original contributions presented in the study are included in the article/Supplementary Material, further inquiries can be directed to the corresponding author.
