# Supplementary material for: “You're listening but you're not hearing”: qualitative exploration of parents' lived experience of paediatric sepsis
Source: Front Pediatr. 2025 Sep 15;13:1655224. doi: 10.3389/fped.2025.1655224 (PMC12478235; doi:10.3389/fped.2025.1655224)
Supplement: Supplementary file 1 [file Datasheet1.pdf]

Supplementary File 1: Standards for Reporting Qualitative Research (SRQR) Checklist

SRQR Item	Description	Location in Manuscript
1. Title	Concise description of nature and topic of study; identifies as qualitative	Title
2. Abstract	Summary including background, purpose, methods, results, conclusions	Abstract
3. Problem formulation	Description of problem or phenomenon studied; significance	Introduction
4. Purpose or research question	Study purpose and specific objectives or questions	Introduction
5. Qualitative approach and research paradigm	Type of qualitative method and theoretical approach	Materials and Methods – Study design and ethical approval
6. Researcher characteristics and reflexivity	Researcher background and potential influences	Materials and Methods – Setting, recruitment and participants
7. Context	Setting/site and contextual factors	Materials and Methods – Setting, recruitment and participants
8. Sampling strategy	How and why participants were selected	Materials and Methods – Setting, recruitment and participants
9. Ethical issues	Ethics approval, consent, confidentiality	Materials and Methods – Study design and ethical approval;<br>Setting, recruitment and participants
10. Data collection methods	Types of data collected and procedures used	Materials and Methods – Data collection
11. Data collection instruments	Interview guides, prompts, tools used	Materials and Methods – Data collection
12. Units of study	Number and relevant characteristics of participants	Materials and Methods - Setting, recruitment and participants;<br>Results
13. Data processing	How data were managed and prepared for analysis	Materials and Methods – Data collection
14. Data analysis	Analytic approach, coding, interpretation	Materials and Methods – Data analysis

15. Techniques to enhance trustworthiness	Strategies like member checking, triangulation	Materials and Methods – Data analysis
16. Synthesis and interpretation	Main findings and interpretations	Results<br>Discussion
17. Links to empirical data	Illustrative quotes or data excerpts	Results
18. Integration with prior work, implications	How findings relate to existing literature and implications	Discussion
19. Limitations	Trustworthiness and methodological limitations	Discussion – Limitations
20. Conflicts of interest	Potential sources of influence or bias	Conflict of Interest Statement
21. Funding	Sources of financial or material support	Materials and Methods – Study design and ethical approval<br>Funding Statement

Reference:

O'Brien BC, Harris IB, Beckman TJ, Reed DA, Cook DA. Standards for reporting qualitative research: a synthesis of recommendations. Academic medicine. 2014 Sep 1;89(9):1245-51.
