# Supplementary material for: “You're listening but you're not hearing”: qualitative exploration of parents' lived experience of paediatric sepsis
Source: Front Pediatr. 2025 Sep 15;13:1655224. doi: 10.3389/fped.2025.1655224 (PMC12478235; doi:10.3389/fped.2025.1655224)
Supplement: Supplementary file 2 [file Datasheet2.pdf]

Supplementary File 2: Interview Protocol

Tell me about when your child first became unwell?
- What were the first signs that you noticed?
- How did you know if this was different to other times they were unwell?
- What was the response that you first got from health professionals?

Tell me about your understanding of sepsis before your child was diagnosed
- Had you ever heard of sepsis?
- Did you know of anyone in your family or community affected by sepsis?

Tell me about your understanding of sepsis now
- How did you gain this understanding?
- Did you receive this information from hospital staff, google, friends, family?
- Do you feel you received adequate education about what sepsis is during your child's admission?
- If you did receive information about sepsis, when did you receive it?

During your sepsis crisis what kind of support did you receive?
- Did friends, family, community, school members make contact and offer support?
- Was this primarily practical support like meals and financial support, or emotional support like being able to talk to and be comforted when upset?
- Do you feel like the support you received met what your needs were at the time?

What type of support would you have liked during/ after your child's admission?
- Do you feel like the support you received was ever tailored to meet the special needs of families affected by sepsis?
- Did you ever want to be put in contact with other families affected by sepsis?

Would you be interested in being involved in a sepsis support group for parents?
- If no, can you talk to me about why you made this decision?
- If yes, can you talk to me about how you imagine this group to work? E.g. Email, social media, website, in person at LCCH, in person at another location/ multiple locations.
- What kind of topics would you like this support group to focus on?

What advice would you give to other parents about sepsis?
- About sepsis education, understanding sepsis
- About hospital life, coping with being in hospital (self-care, talking with medical teams)
- About receiving support
- About adjusting to going home
- About adjusting to having a child with a disability
- About being a bereaved parent or family member
- About your overall experience of sepsis and your families overall experience of sepsis

If a campaign was developed to create more public awareness of sepsis, can you give me and ideas about what could be included in this?

If a group was developed of family members affected by paediatric sepsis that we consulted with during the development of such campaigns, or developments in sepsis research, would you be interested in being a part of this?

Do you want to know about ongoing research and developments in sepsis treatment?
If yes, how would you like to receive this information?
