# Supplementary material for: “You're listening but you're not hearing”: qualitative exploration of parents' lived experience of paediatric sepsis
Source: Front Pediatr. 2025 Sep 15;13:1655224. doi: 10.3389/fped.2025.1655224 (PMC12478235; doi:10.3389/fped.2025.1655224)
Supplement: Supplementary file 3 [file Datasheet3.pdf]

Supplementary File 3: Illustrative Comments

**Rupture of life as we knew it**	
Recognition that something about their child's condition had changed or was different this time	*"My husband thought he was resting and getting better. But... I said, 'something's wrong, that's not right'. So, we called the after-hours [medical service] and, then it was all on, the ambulance came, and they took him in." – Bereaved Parent 5*
	"I said to my husband 'it's just not right, something's just not right'." – Survived Parent 1
	"She slept most of the day and I kind of just went, 'let her just sleep and rest and get through it' and in the back of mind I'm [thinking], 'we are going to the hospital tomorrow there is something not 100%'." – Bereaved Parent 3
	"And I just said, 'I just think that there is something worse wrong'." – Bereaved Parent 1
Preparation for multiple potential life trajectories	*"And they started to warn me that he might lose limbs, he might need this, he might need that and was trying to prepare us for if he survives." – Bereaved Parent 1*
	"Initially in ICU we were asking the doctors you know, how much is she going to lose? And they were like you know probably fingertips, toes, you know, but the blackness just kept, kept going up her arm. And they wrapped them at some point so you couldn't see." – Survived Parent 1
	I thought she just needed some fluids, and then when they said, 'okay the team is coming from [tertiary hospital]', I went 'okay'. And I'm preparing myself [thinking,] 'okay we're going to be in [tertiary hospital] for a couple of weeks, that's fine, that's all right, that's just what it's going to be, she might be there for quite a few weeks, but we can deal with that'. And then over the next few hours her fingers started [showing signs of necrosis]."– Bereaved Parent 3
	"And he sat us down and he told us that it was pretty serious, and that she was on the scale for liver transplant, potentially, and kidney failure and all the rest of it. Like it was pretty heavy going." – Survived Parent 4
	"If he had survived, he would have lost both of his legs." – Bereaved Parent 4
	"Like we were sitting in a room with 15, 16 doctors, and I think probably 10 of them were telling us that the arm needed to go. But when you got your 15-year-old daughter in the bed pleading with the Plastic Surgeon and his team, 'don't take my arm' [pauses, crying]… it was incredibly difficult." – Survived Parent 4

	"You know we did all the scans, it started off he's getting a little bit better, he's not, he's brain damaged, then they told us on the Thursday night that he's not going to make it." – Bereaved Parent 5
	"Eventually they said we can wake her up six days later. And we didn't know what we were going to have mentally." – Survived Parent 1
Importance of describing who their child was before sepsis, and the stark contrast between their life and the deterioration they experienced	*"My son was a very robust, big, seemingly healthy child... and eight hours later we lost him. Cardiac arrested three times... it was too late." – Bereaved Parent 1*
	"She was healthy, gorgeous, just started walking… she was okay on Sunday, and gone on Thursday." – Bereaved Parent 3
	"He was sick on the Monday, he died on the Friday, and he was the sweetest 16-year-old you'll ever meet. I really was lucky." – Bereaved Parent 5
	"And she was healthy, no issues beforehand with her, um at all. Yeh, so, she had her arms amputated on the 10th of November. And her legs we waited for until the 3rd of January." – Survived Parent 1
	"He was a big boy, he was 6ft tall you know, and a wide big lad, he's never been sick in his life... he had a golden staff, bilateral pneumonia, influenza, and obviously the sepsis which is the top of everything." – Bereaved Parent 4
Assumptive worlds were shattered	*"You're gripping on to, you know, babies don't die, not in 2018, not in Australia." – Bereaved Parent 2*
	"I didn't know anything could kill someone so quickly… I didn't know there was anything like that out there that could kill you so quick." – Bereaved Parent 7
	"He probably had at least eight or nine admissions before we lost him, but he always recovered, right?" – Bereaved Parent 1
	"I thought septic shock came after a burst appendix, and after something that happens to you in hospital, you know you get it after an infection normally in hospital, not from the flu, or not from nothing, this appears to have come from nothing." – Bereaved Parent 7
	"That's what everybody tells you, you recover from pneumonia, you recover from the flu." – Bereaved Parent 1

**Navigating in the Dark**	
Unaware of sepsis prior to episode	*"In our situation, yeah, completely naïve to the word 'sepsis'. And, um, my husband had studied one year of medicine." – Bereaved Parent 2*
	"We've never heard of sepsis, I'd never heard of it... I'd never heard the word." – Bereaved Parent 7
	"I didn't know anything could kill someone so quickly, like we've never heard of sepsis. We've heard of meningococcal, and you know how quick meningococcal is, that can happen in a day, but there's a rash. I didn't know there was anything else out there like that, that could kill you so quick." – Bereaved Parent 7
	"She had sort of said, 'I hope this doesn't become septic'. But I didn't have a clue what they were talking about. I know nothing about sepsis at that point." – Survived Parent 4
Inconsistent responses from health professionals regarding initial illness and safety-netting	*"She [daughter] sort of tried to stand on her legs, and she couldn't. The doctor called another doctor in and um, didn't really give me much interest. I think the general comment was 'oh, you know it's Influenza, we've had a lot of that this season, just go home and rest'." – Survived Parent 1*
	"And he died over three, he was sick three days before similar scenario, GP, back to GP, 13 HEALTH, back to GP." – Bereaved Parent 2
	"Took him home, they [medical team] said, 'go home', I said, 'I'd prefer we admit him now' and they are like, 'no, no go home'." – Bereaved Parent 1
	"So we rang the hospital again not knowing that you can't really ring a hospital and talk to somebody in the Emergency Department. So they said, 'oh look we will put you through to [another healthcare provider]'. And it was quite a long conversation going through her symptoms and my husband even said, 'I do not want to be the parent who has a child with, you know, do you think it's Meningococcal?' because that is what we know about, you know we were so worried. And she said, 'you've been to the hospital, just rest, they have given you that diagnosis' sort of thing and my husband's like you know I don't want to be the parent who doesn't do enough." – Survived Parent 1
	"She started getting temperatures and her Mum knew what was going on and she questioned [medical team] she said, 'look she's getting temperatures and I think it must be an infection'. We were told, 'no-no, that's normal post-operative reactions'. And then they gave [child] some Gentamicin or something which settled her temp down for a day and they actually sent us home." – Survived Parent 4

Received limited sepsis information and ineffective communication during critical illness phase	*"It's just a blur like nobody explained what it could be. We didn't, I didn't comprehend how sick she was at all." – Bereaved Parent 3*
	"You're listening but you're not hearing." – Bereaved Parent 5
	"I'm incredibly thankful that I am married to [wife's name] and she knows what's going on in this industry. Because I didn't really have a clue what was going on, and it was, it's pretty scary." – Survived Parent 4
	"In a way it's complex for people to understand maybe. But you know they didn't explain at all, I didn't get a brochure, I didn't get, I got nothing." – Survived Parent 1
	"They said right from the second presentation sepsis was in the notes, but we had no information about that. They knew what they were looking for, and we knew nothing about that, they didn't tell us that. So, they were tiptoeing around us rather than telling us the information. I'm somebody that because of my profession I want to know the facts, I have to know the facts. And the doctor just wasn't giving us that information." – Survived Parent 2
	"I think it was, it was something like ten days [after child had died] before I heard [the word] sepsis." – Bereaved Parent 2
	"You're not there, you're sort of hovering, you're there, but you're just, you're helpless." – Survived Parent 1
	"It's a whirlwind hey, I remember, I think back now and it's like watching a movie." – Bereaved Parent 5
Deterioration due to sepsis is rapid, visual, shocking, and traumatic	*"At the time I still thought he would live, I didn't think he was going to die. I even said, 'Is my baby going to die?' [Nurse replied,] 'No sweetheart, your baby is not going to die, he has improved, he is getting better'. And then I shouted, 'Somebody needs to look at my baby, somebody help me' [crying, gesturing as if holding child]. He died." – Bereaved Parent 2*
	"I was walking back, and I heard them saying her heart had stopped. I remember just standing there in ICU and I yelled at my husband 'we are losing her' and they were pumping her chest." – Survived Parent 1
	"So we forced him into the car. And uh, he walked into the hospital here, and um I think as soon as he walked in the triage nurse, he was, you know well, that was the last time we spoke to him [pauses]. To, uh, to my boy [pauses, crying]." – Bereaved Parent 4

	"I think what connects all of us is this traumatic and short period of time in which we've gone from healthy, to either impact the rest of their life or not here at all. No more, nothing." – Bereaved Parent 1
	"I think it's also, sepsis is very visual [multiple people agree verbally and non-verbally] they don't look the same, and they, you know, so I think that's, you live with these images in your mind, and I don't know if that's different for people who lose children in different ways." – Bereaved Parent 1
	"He had an ear infection, he was on antibiotics, and then he died." – Bereaved Parent 2
	"And when I got up in the morning she was just grey, and she wasn't moving much." – Bereaved Parent 3
	"This is him yesterday playing football and doing everything. And here he is today. And that was from 9 o'clock in the morning to 3 o'clock in the afternoon. And the next morning he was black [describing tissue necrosis]." – Bereaved Parent 4
	"Then I notice his lips blue, and that's it, we go. So he was at the GP around 9am or 10am, and this is about 2pm. So I think by the times of ER [Emergency Room] and everything, and intubating if you look at all of that, that's about maybe 2:30pm, the ECMO might be about 3-3:30pm." – Bereaved Parent 7
	"Like there's days when, when I am at work, and driving my dozer pushing dirt and all of a sudden I'm in tears, just thinking about [child's] episode." – Survived Parent 4
	"Um, but obviously she is a quad amputee, so she's lost her below elbow and below knee." – Survived Parent 1
	"And you know [they placed him on] ECMO, and then they did two ECMOs, and oh god, it was just so traumatic." – Bereaved Parent 7
	"I've blocked a lot out, and now two years later it's just starting to come in, because I couldn't deal with it." – Bereaved Parent 3
	"His body had blown up like a balloon." – Bereaved Parent 7
PICU is an overwhelming and unfamiliar environment, contributing to overall distress.	*"You want your family close, and you need support but there's nowhere to eat, or sleep, or where are the toilets if you've never been in a hospital situation, where can I shower?" – Survived Parent 1*
	"ICU is hell on earth and I never want anyone else ever to be there [becomes upset, crying]." – Survived Parent 1

	"I wish I had [the nurse's] advice earlier about talking to him more. Well, I did after that, but I should have straight away." – Bereaved Parent 7
	"I really concentrated carefully on the aesthetic in PICU. So, I had to minimise the beeping at night, I'd turn the lights down, I put things over his eyes at night, I'd try to get everyone to be a bit quieter. And then in the day and I'd play quiet night music, and I'd put on the cricket on for him in the morning, and then a bit of music in the afternoon." – Bereaved Parent 7
Sepsis is complex and difficult to understand	*"But I was talking with other people about it, about whether or not I think I'd recognise it again. And I like to think that I could, but maybe I wouldn't, you know?" – Bereaved Parent 4*
	"Can you catch sepsis again, that's something I still don't know?" – Survived Parent 1
	"And it took a while for us to understand, but like when I was ringing my Mum last night she still doesn't understand what it is." – Survived Parent 1
	"So like, what really is that? [what really is sepsis?]" – Bereaved Parent 6
	"Well I probably still really didn't fully understand it [referring to sepsis]. I mean even when all of that happened, like I was, well she got fully resuscitated that day in intensive care and all the rest of it. I mean you know that something serious is going on but I guess I still didn't take the time to get a full understanding of exactly of what sepsis meant." – Survived Parent 4
Experiences of isolation during and after child's sepsis	*"Because sometimes at home I feel like nobody really understands what we went through, or what sepsis is. And I'll just be driving with him down the road and I'll remember what happened to him and absolutely bawl my eyes out." – Survived Parent 3*
	"Like I said, I've got some friends I've grown up with my whole life and they still can't get it in their heads. And I feel like slappin' them, you know?" – Bereaved Parent 4
	"Because you lie in bed sometimes and go, 'I've got no one to talk to, I've got no one." – Survived Parent 1
	"There are no support groups out there." – Survived Parent 1
Isolation experienced by rural and remote families amplified due to having access to even fewer supports	*"There's not even any child loss support groups in my [hometown] let alone sepsis child loss support groups." – Bereaved Parent 7*

Grief experienced by families bereaved by sepsis is unique, complex, and isolating	*"I think what I found was after we went home, and after everyone else left, I've never felt so isolated, and I felt like I was the only parent in the world who's lost a child to sepsis. And I went online looking for groups, or other bereaved parents and I couldn't find anything." – Bereaved Parent 3*
	"Right away you're looking for that support and I use to go to a lot of these initially [referring to general bereavement support groups] but I didn't want to know about all these other ways you could lose your child." – Bereaved Parent 1
	"I still had him after he died, he was here [gesturing to holding child in arms]. He was, my baby, oh gosh [crying]." – Bereaved Parent 2
	"And I thought 'wow, she is still alive, she goes to work, she has other children, she has created a life, she has continued to survive'. You know like, um, I think that was really big [referring to being connected with another parent bereaved due to sepsis]." – Bereaved Parent 2
	"Yeah, I would really love to still find out more about it too [referring to understanding what sepsis is]." – Bereaved Parent 6
	"Like afterwards too I was like 'oh man I would have kept him, even if he couldn't walk, couldn't talk' I would have nursed him." – Bereaved Parent 5
	"Mind you I still feel the struggle. I've never opened my son's dresser to this day. It is perfectly formed with all of his clothes. I can't open it. But we do survive, we choose, we make choices I suppose, and we honour our sons." – Bereaved Parent 1
Autopsy processes (both coronial and hospital) are distressing and complicated to navigate	*"Just before we were about to say goodbye they said, 'they're going to do an autopsy, the coroner will take over'. And they had to do an autopsy, and we just said no, the last thing this little girl needs is that." – Bereaved Parent 3*
	"When a child dies in hospital and they don't know why the coroner and police come, and you talk to the police [crying] and they interview you, they interviewed my husband, they interviewed my sister-in-law." – Bereaved Parent 2
	"You know his Mum didn't want to have an autopsy you know she doesn't want to have her baby… [crying]." – Bereaved Parent 4

	"We had trouble getting his autopsy report and that's uh, quite a bit of a run around, that frustrated me quite bit trying to get onto the right people. I think we could speak to them and they'd say, 'oh you gotta go to the coroner's' and we'd say, 'it's not a coronial, it's not a coronial death'. It gave us quite a bit of a headache to be able to sort it out, it took quite a few months, I think it was 4-5 months before we could get a copy of it." – Bereaved Parent 4
**The weight of 'what if'**	
Guilt, regret and anguish that there was a missed opportunity to change child's sepsis experience and outcome	*"That's always in my mind and will never go away. If we had of done blood tests on that day, rather than waiting to the next day to go to the hospital, would the outcome have been different? And the outcome is what it is. But that's one thought I have struggled with." – Bereaved Parent 3*
	"But I have guilt, it's a stupid guilt, but I remember before we lost him, I whispered in his ear, 'if it's too hard [son's name] you can go'. And I regret it, I said 'what if I had told him to fight, would he have listened?' Like, so that's a regret I have, that I told him it was all right to go. Well sure enough on Friday he left. And I just thought, if anyone's going to tell him he can go it's his Mother. But I regret it, I still regret it, in a stupid way I regret it." – Bereaved Parent 5
	"My husband regrets it too because I wasn't here, he goes, 'oh if your mum was here she might've', but we listen to the doctors because you think they, you know, [they say,] 'go home and rest'. And you know, you trust them, so I tell my husband, 'don't blame yourself'." – Bereaved Parent 5
	"And um, yeh I dunno I guess, I wish I had let his Mum Mother him a little bit more, as they were getting older them boys and, then maybe, maybe we would have had a different outcome." – Bereaved Parent 4
	"But you know, I still wonder about blood tests or if he had been treated for Mycoplasma and had had the right antibiotics would he have been here?" – Bereaved Parent 1
	"But then you know, I just wonder about why we didn't do the PET scan earlier." – Survived Parent 4
	"I should have made a scene, I should have made him not play, and I should have just made a scene, but I didn't, I just let him do it." – Bereaved Parent 7

	"And I thought maybe I should go to him, you know, I thought about going to him, and I didn't, I didn't go to him [pause] so I left him there, and I should have gone to him. I should have gone and got him and gone to hospital. That's what I should've done. But I didn't do that." – Bereaved Parent 7
Barriers to advocating and having parental concerns validated	*"She [the Doctor] said 'go home and rest'. Okay you know, [I thought] 'well they know what they are talking about, I don't, off you go, we will go home'. We were so worried." – Survived Parent 1*
	"They [medical team] said, 'just take him home, he'll be fine, just watch and see how he goes'. I actually worked overtime because I thought, 'nup, the hospital sent us home, he's fine, he'll be fine, there's nothing to worry about, he'll be okay'." – Survived Parent 2
	"[I thought] I won't take them back because I don't want to bother them [the medical team], they said they're okay." – Survived Parent 2
	"But I walk away think my god how many parents lose their kids because the parents know no better as well? Haven't got that knowledge in the background to back up their argument. Yeah, they might complain to the doctors, but they might not actually know what they're complaining about." – Survived Parent 4
	"You worry about being paranoid parents. Rather be paranoid and have them be perfectly healthy than…" – Bereaved Parent 1
	"There were all these warning signs, but I'm like [pauses, crying], sometimes you just become a mess, you don't know what to do, you can be so strong in some situations but then when it's your own kid you're just hopeless." – Bereaved Parent 7
	"Look I'm from [rural area], so I just thought you know I'm in [metropolitan area], they gotta be right you know, when you're in a bigger place." – Bereaved Parent 4
	But if I knew the term I mean well before, a number of us went back and forth between doctors, looking at our child going yeh I know this is not getting better, to be able to ask somebody 'can you check for sepsis?'." – Bereaved Parent 1
**Call for Change**	
Finding meaning and purpose after their child's sepsis	*"I think not long after he passed, they had quite a lot more success on putting some other kids on two machines [ECMO]. So, I feel a bit of joy in that. Maybe the opportunity to put him on that helped another couple of children." – Bereaved Parent 4*

	"I didn't want the autopsy at first, I was like, 'no, look at him, he's been through enough'. And then, we had a talk [my husband and I] and we said well you know that's his nature. And we wanted to give all his organs, but we couldn't, they were no good, so we said do the autopsy in case it can save another kid. But it was hard, it's not an easy decision." – Bereaved Parent 5
	"I just felt. I can't bring him back, and hope that maybe somebody here has learnt their lesson because there's an awful lot of investigations going on here." - Bereaved Parent 1
	"After my son died I had a lady Facebook me, it might have been two weeks ago, [crying] sorry, and she said, 'if I hadn't seen his story', it was on the news, 'if I hadn't seen his story I probably would have just let my kid be home sick with the flu'. She goes, 'but something kept playing in me'. She took her in and she had sepsis. She survived, she survived." – Bereaved Parent 5
	"The stuff they are doing up home there [referring to raising awareness of sepsis], I like to think that, you know it's good for our young kids. Even if the younger generation can familiarise themselves with the signs of what can happen and you know?" – Bereaved Parent 4
	"So many people have heard of Sepsis up here now, just because of the work that we are doing." – Bereaved Parent 7
	"I have had fifteen years to adapt to losing my son who was two and a half, and um, jeez, in fifteen years I have been quite involved in his keeping his name perpetuated through a fund." – Bereaved Parent 1
	"Ours is sepsis awareness and child survivor support [referring to organisation they developed after death of child from sepsis]." – Bereaved Parent 7
	"And we raised some money and contributed to some research." – Bereaved Parent 2
	"So how do we, because it all comes down to money, basically for funding, you know to get awareness, awareness campaigns, and to see things change in hospitals. Who, how can we get the government to fund this? We need to do something about it, do we do a petition or something?" – Survived Parent 1
Quality family-centred care is required to assist familial coping with emergency and PICU environments	*"We got to take him outside on the balcony, and… [pause, crying], I think it was four thirty in the afternoon, so the sun comes in, and he got to be outside to pass. So, we was appreciative of that. The extra effort that everyone here went to to help his Mum and myself and our other son." – Bereaved Parent 4*

	"The staff here were amazing with our other daughter, so good with her, I didn't have to worry." – Bereaved Parent 3
	"We were sleeping in the waiting room, we weren't leaving, and they didn't force us to leave. And we literally brought mattresses in and we slept, and there was always someone by [son's] bedside. And then we had like a little service, and everyone got to say goodbye." – Bereaved Parent 5
	"We stayed in the Ronald McDonald House briefly, it was good to have that there." – Survived Parent 1
Need for paediatric sepsis-specific family support network	*"And you just want to talk, and your friends try and help, and your family tries to help, but you just want to talk to someone who just goes, 'I get it, I get it'. Even just sitting here, I feel like I'm not the only one, for the first time in two years." – Bereaved Parent 3*
	"So if it was improving the service, even though after the fact, it would be having some sort of support network of parents who have been through it that you could just ring and talk to." – Bereaved Parent 3
	"I like the idea of a siblings day, I think [sibling], because now she's on her own, I think as she gets older she will need to [pauses, crying], you know, I just think make an event, a family BBQ in the park, away from the hospital but you still have that commonality." – Bereaved Parent 3
	"And well, connecting with [another parent whose child had sepsis] it was a blessing, it was many years since I had already lost my child by the time I met you, but I spoke about it to you, like I hadn't spoken to anyone about it." – Bereaved Parent 1
	"Our new normal was, someone else knew what we were talking about." – Bereaved Parent 5
	"You feel heard [when talking with other parents of children with sepsis]." – Bereaved Parent 3
	"Someone gets it." – Survived Parent 1
Need for education of public and health professionals on sepsis signs, symptoms, management, and role of parental concern for early detection	*"Trust your gut. You know your kid. If you know that there's something wrong with your child do not leave that hospital." – Survived Parent 2*
	"I guess speaking as an uneducated medical person, I would say back your gut instincts. Like as parents, you know your child better than anyone else. You know what their pain thresholds are and all that sort of stuff. And like you

	pick up on your kid's mood and all that sort of stuff. Like, I guess, yeah that's probably the biggest thing for me - and ask the stupid question." – Survived Parent 4
	"I'd like to see public awareness campaigns so that it is as well-known as Meningococcal, but I'd also like to see this rolling out to GPs and hospitals so that they will err on the side of caution if you suspect that there is a bacterial infection whether they are two years old or twelve years old give them a blood test and be proactive rather than waiting." – Bereaved Parent 3
	"I think it's making the information available, well before, well before, you know where you can go and ask the doctor could, back and forth three or four times, 'could they have sepsis?'." – Bereaved Parent 1
	"I think it's just educating parents, if you know that's there's something wrong with your child, regardless of what the doctors say at the hospital, if you're not happy, seek medical attention." – Survived Parent 2
	"I would just say to all the parents to get to know what sepsis is all about really, you know? Because if they hear the doctor telling them the child or something has sepsis, go and find out more about it." – Bereaved Parent 6
	"It's a word that kills most people in the world, but no one has ever heard of it, you know?" – Bereaved Parent 7
Importance of safety netting to educate and empower parents of unwell children to re-present if concerns continue	*"You might not have all of those symptoms because everyone has different symptoms, you might have some, but if you're worried come back, you're welcome to come back." – Survived Parent 1*
Need for timely, trauma informed education on sepsis and escalation pathways	*"I think a pamphlet would have been good for me, like in that time when we were first in ICU, I might have gone back there [to the accommodation] in quiet times and looked at it then away from the bed. I think I would have liked to have known." – Survived Parent 1*
	"If they're considering that it is sepsis, if they try and tell a parent and that's not getting through then perhaps a pamphlet would be a good option because that way when a parent has got a moment of time to breathe and go, 'okay well I need to look at what's actually happening with my child' they can read something in their own time." – Survived Parent 2
	"I just think as far as the Ryan's Rule [name of escalation pathway in health organisation] thing is concerned, I do think at some point someone from the hospital needs to sit down with every parent, like in that initial day or two or in initial booking in phase and sort of go through it a bit better with them and make it a bit clearer." – Survived Parent 4

	"I'd never heard of Ryan's Rule [name of escalation pathway in health organisation], and I think that's another thing, because you know your child, you think something's not right, whatever it is, like maybe not sepsis, maybe there's other things that I'm just not aware of. You use that, and they have to act then. That's another tool that I just don't think parents are aware of exists." – Survived Parent 1
Bereaved parents, and parents of children who survived, have different ongoing support needs to one another	*"But the difference is that we've lost our children, our need is our grief and then to process that. We've all gone through a similar trauma. But where our life goes, your life, and your challenges now, they are just very different to what ours are going to be." – Bereaved Parent 1*
	"I think we are travelling different journeys." – Bereaved Parent 7
	"I reckon it might be better separate. I'm not being mean or anything I just, sorry. I just think you'd have more people to bounce off because you know, you might be going through what you're going through now and to help". – Bereaved Parent 5
	"It might be better to be separate, you know because yes he has been impacted by sepsis, and he will have ongoing issues because of what happened to him, but there is still an element of guilt because I still have my baby." – Survived Parent 2
Rural and remote families have unique and additional needs with regards to accessing healthcare and support	*"And I want to fly the flag for rural and regional Queensland too. It would be wonderful if, regardless of where you live in Queensland you felt safe." – Bereaved Parent 2*
	"Yeah. I probably would [like to access sepsis-specific family support]. It's just my distance or where the location would be and that." – Bereaved Parent 6
